# Editorial: Above-belowground interactions involving plants, microbes and insects

**DOI:** 10.3389/fpls.2015.00318

**Published:** 2015-05-27

**Authors:** Ana Pineda, Roxina Soler, Maria J. Pozo, Sergio Rasmann, Ted C. J. Turlings

**Affiliations:** ^1^Laboratory of Entomology, Wageningen UniversityWageningen, Netherlands; ^2^Department of Terrestrial Ecology, Netherlands Institute of Ecology (NIOO-KNAW)Wageningen, Netherlands; ^3^R&D Microbiology, Koppert Biological SystemsBerkel en Rodenrijs, Netherlands; ^4^Department of Soil Microbiology and Symbiotic Systems, Estación Experimental del Zaidín, CSICGranada, Spain; ^5^Laboratory of Functional Ecology, Institute of Biology, University of NeuchâtelNeuchâtel, Switzerland; ^6^Laboratory of Fundamental and Applied Research in Chemical Ecology, Institute of Biology, University of NeuchâtelNeuchâtel, Switzerland

**Keywords:** plant defenses, plant-insect interactions, beneficial microbes, root herbivores, signaling pathways, multitrophic interactions, phytohormones, induced resistance

“Integration” has become a central theme in plant research, and this concept has greatly contributed to the exciting recent advances of our understanding of plant interactions with their environment. Plants in nature interact with a plethora of organisms, from microbes and insects through vertebrates to other plants. While some organisms are detrimental, others have mutualistic interactions with plants. Whereas, plant responses to all these organisms were initially studied separately for aboveground and for belowground domains, nowadays it is accepted that plant interactions belowground orchestrate a cascade of events that affects the interactions of plants with organisms that live aboveground, and *vice versa*. In parallel, it has become evident that the plants' interactions with both mutualists and antagonists are governed by often similar phytohormonal and metabolic responses, which starting at the molecular level, shape the ecological interactions that take place in natural and agricultural systems. Beyond the fundamental interest in these interactions, a comprehensive understanding of how plants integrate their responses to microbes and insects below-aboveground, will also contribute to tackle applied concerns such as the development of sustainable crop protection strategies, and the development of novel crop varieties for increasing yield and performance. Integrating plant responses to beneficial and detrimental organisms, from microbes to insects, and using integrative approaches that range from the molecular changes within the plant to the ecological interactions taking place among organisms, will provide a more representative view of the multitude and complexity of interactions in nature. With this Research Topic, we aimed at being integrative, by proposing a wide array of themes, spanning papers dealing with the molecular bases of above-belowground plant-insect-microbe interactions, to more ecological and large-scale work.

The integration of several approaches to study belowground interactions is the focus of a significant methodological contribution of Campos-Herrera et al. (2013), where the authors review recent advances in molecular, chemical, and spatial techniques for studying belowground ecology. Particularly, they expand on how molecular tools could be developed and used to measure diversity and distribution of soil nematodes, and propose how can it be exploited in biological control. Nonetheless, the steep road necessary to uncover mechanisms of interaction happening in the soil has just started, and these authors nicely outline how collaboration with different fields will be continue to be necessary in future research belowground. In a different framework, integration of below-aboveground research is what prompted Balmer and Mauch-Mani (2013) to pose the intriguing question of why plant organs such as leaves and roots mount such different defense responses against their microbial and herbivorous attackers. They concluded that roots and shoots use different defense machinery, and using an evolutionary and ecological approach, this could be explained by feedback mechanisms between plants and the microbial communities.

It is estimated that all plant species establish symbioses with microbes. Most of these so-called beneficial microbes are located belowground in the rhizosphere, where vast numbers of different microbial species reside. The plants as we perceive them aboveground are the result of millions of years of coevolution with these beneficial microbes. From this perspective, Van Geem et al. (2013) discuss how above-belowground interactions across several trophic levels may have been driving genetic variation in plant traits. As a consequence, plant-insect and plant-pathogen interactions aboveground would have been indirectly (and secretly) shaped by symbiotic associations belowground. Focusing on beneficial microbes belowground, Pangesti et al. (2013) discuss the how moving from addressing interactions between plants and single microbial species to a community approach could move forward our understanding of the complex soil ecosystem. Their major take-home message is that following this community approach below and aboveground in combination with the understanding of induced systemic resistance (ISR), will increase the reliability and durability of the application of beneficial microbes. A long-standing question in the studies of microbes and insects is: what is driving the context-dependency of the effects of beneficial microbes on insects? Barber et al. (2013) provide an interesting example where different mycorrhizal strains impact both herbivores and pollinators in a way that depends both on the mycorrhizal fungi and insect identity. Vannette et al. (2013) move a step further, and show that plant genotype can also be a crucial factor in these interactions. They point out that the effects of mycorrhizal fungi on, for instance, cardenolides, the main defensive compounds of milkweeds (*Asclepia* spp.), vary with plant species. Most fascinating is the fact that this seems to be explained by the phylogenetic origin of those species, and it appears that the tradeoff between plant growth and defense can be mitigated by mycorrhizal inoculation. Working with a different group of soil-dwelling organisms, Wurst (2013) explores the underappreciated role of the indirect linkage between detritivores and plants in plant-mediated above-belowground interactions. Indeed, even though detritivorous soil fauna is ubiquitous and present in high abundances, it is still highly underrepresented in above-belowground studies. The few existing studies in this regard highlight both positive and negative effect of detritivores on plant resistance, thus currently impairing generalizations on the topic.

Phytohormones, and particularly jasmonic acid (JA) and salicylic acid (SA), have been shown to orchestrate induced plant defenses to herbivory and pathogen infection. Nevertheless, how plants integrate their responses when under multiple below-aboveground attackers is not yet fully understood. For instance, the role of JA signaling goes beyond effects on herbivores, and with their experimental research Constantino et al. (2013) highlight the importance of belowground ISR on aboveground tissues. In a detailed experimental paper, they show that the JA-biosynthetic gene *LOX3* of *Zea mays* is a negative regulator of ISR, and down-regulation of its expression in the roots by the beneficial soil fungi *Trichoderma virens* is required for triggering ISR on the leaves. Interesting is also their finding that the xylem sap contained the responsible factor for these effects. Along the same lines, Shavit et al. (2013) showed how JA and SA signaling pathways may mediate the positive effects of plant growth-promoting rhizobacteria (PGPR) on whiteflies aboveground. They observed a decrease of the induction of JA and SA-marker genes by PGPR, and propose that changes in the microbial community of the rhizosphere may trigger this effect.

Other hormones, which are traditionally considered to be involved in plant growth and development, are also gaining attention as possible regulators of plant interactions with microbes and insects (Pangesti et al., 2013). These hormones include auxines, cytokinines, or abscisic acid (ABA), with the latter being known for its crucial role in responses to abiotic stress. In a comprehensive experimental paper, Vos et al. (2013) show that ABA is an important regulator of herbivore-induced plant resistance. More specifically, a functional ABA signaling pathway is required to mediate the effect of previous herbivory on the reduction of subsequent herbivore performance and the associated induction of JA-regulated responses. These studies substantiate the fact that plant signaling pathways can regulate responses to all kinds of stresses, from abiotic to biotic, from microbes to insects, and from mutualistic to detrimental interactions.

One major concern this decade is facing, is how climate change may alter plant-mediated interactions among root and shoot associated organisms. Little is known, and almost no empirical evidence has been generated on this matter. In a perspective article McKenzie et al. (2013) explore this relevant issue, highlighting the importance of the belowground domain when attempting predictions on the effects of climate change on plant-insect interactions aboveground. Providing rare experimental evidence, Ryalls et al. (2013) show how elevation of temperatures and CO_2_ can influence below-aboveground interactions between plants, microbes, and insects. Specifically, they show that elevation of temperatures and CO_2_ had no effects on aphids, whereas had contrasting effects on weevils that potentially occurred through changes in root nodulation patterns. By predicting plant species distributions along environmental gradients, Pellissier et al. (2013) explore the long-standing question of what drives plant species assemblages in space. The underlying hypothesis of their work is that plant and fungi mutually influence their spatial distribution. They show that by including information on fungal abundance and identity, it is possible to significantly improve the predictive accuracy of plant species distribution models, especially for plant species occurring at high elevations. Indeed, colonization of high elevation soils should be facilitated by the presence of mycorrhizal fungi, but this awaits experimental confirmation.

One another major contemporary challenge in plant research is how to achieve an economically, sustainably, and environmentally-sound increase in the production of high quality foods. Fundamental knowledge of how plants interact with their attackers and beneficials provides the basis of modern agriculture. An example of this is presented in the experimental article by Pierre et al. (2013), in which it is shown how the application of the phytohormones SA and JA on broccoli plants decreases the incidence of several devastating insects pests, both below- and aboveground. Interestingly, these effects were not mediated by glucosinolate induction, and they only affected specialist herbivores and not generalists, a rather uncommon pattern that merits future attention.

A fascinating and very promising topic, but still scarce in the literature, is the fact the some beneficial rhizobacteria also have a direct antagonistic effect on root herbivores. The review by Kupferschmied et al. (2013) provides a state of the art of this topic. Insecticidal properties of *Pseudomonas* bacteria are proposed as a novel biological tool to tackle important root herbivore pests, with the advantage of a high persistence due to their adaptation to the life on plant roots. Along the same lines but in a wider context, Orrell and Bennett (2013) take on the challenging question of how fundamental knowledge on plant-mediated above-belowground interaction can be exploited in practice to enhance crop productivity. They suggest that the manipulation of above-belowground interactions may help to reduce losses to pests and increase crop yield by either implementing better intercropping strategies, by selecting for specific plant traits that mediate beneficial above-belowground interactions, or by breeding mutualistic organisms to enhance the benefits of the interactions.

In this Research Topic we aimed at highlighting the need for a good understanding of plant-mediated below-aboveground interactions between insects and other organisms, and that this requires the integration of both mechanistic and more holistic, ecological approaches. This diverse set of excellent contributions has also the merit to highlight that nature as we observe it aboveground, is the product of a diffuse co-evolutionary history between plants and a multitude of other organisms, living above- and belowground. It is therefore not surprising that roots are now being considered the key of the second green revolution, and the study of their interactions with organisms living in the rhizosphere should be now fully integrated to boost food production in a sustainable manner.

## Conflict of interest statement

The authors declare that the research was conducted in the absence of any commercial or financial relationships that could be construed as a potential conflict of interest.

## References

[B1] Balmer D. Mauch-Mani B. (2013). More beneath the surface? Root versus shoot antifungal plant defenses. Front. Plant Sci. 4:256. 10.3389/fpls.2013.00256 23874350 PMC3709096

[B2] Barber N. A. Kiers E. T. Hazzard R. V. Adler L. S. (2013). Context-dependency of arbuscular mycorrhizal fungi on plant-insect interactions in an agroecosystem. Front. Plant Sci. 4:338. 10.3389/fpls.2013.00338 24046771 PMC3763484

[B3] Campos-Herrera R. Ali J. G. Diaz B. M. Duncan L. W. (2013). Analyzing spatial patterns linked to the ecology of herbivores and their natural enemies in the soil. Front. Plant Sci. 4:378. 10.3389/fpls.2013.00378 24137165 PMC3786222

[B4] Constantino N. Mastouri F. Damarwinasis R. Borrego E. Moran-Diez M. E. Kenerley C. M.. (2013). Root-expressed maize lipoxygenase 3 negatively regulates induced systemic resistance to *Colletotrichum graminicola* in shoots. Front. Plant Sci. 4:510. 10.3389/fpls.2013.00510 24391653 PMC3867115

[B5] Kupferschmied P. Maurhofer M. Keel C. (2013). Promise for plant pest control: root-associated pseudomonads with insecticidal activities. Front. Plant Sci. 4:287. 10.3389/fpls.2013.00287 23914197 PMC3728486

[B6] McKenzie S. W. Hentley W. T. Hails R. S. Jones T. H. Vanbergen A. J. Johnson S. N. (2013). Global climate change and above- belowground insect herbivore interactions. Front. Plant Sci. 4:412. 10.3389/fpls.2013.00412 24155750 PMC3804764

[B7] Orrell P. Bennett A. E. (2013). How can we exploit above–belowground interactions to assist in addressing the challenges of food security? Front. Plant Sci. 4:432. 10.3389/fpls.2013.00432 24198821 PMC3812866

[B8] Pangesti N. Pineda A. Pieterse C. M. J. Dicke M. Van Loon J. J. A. (2013). Two-way plant mediated interactions between root-associated microbes and insects: from ecology to mechanisms. Front. Plant Sci. 4:414. 10.3389/fpls.2013.00414 24167508 PMC3805956

[B9] Pellissier L. Pinto E. Niculita-Hirzel H. Moora M. Villard L. Goudet J.. (2013). Plant species distribution along environmental gradient: do belowground interactions with fungi matter? Front. Plant Sci. 4:500. 10.3389/fpls.2013.00500 24339830 PMC3857535

[B10] Pierre S. P. Dugravot S. Hervé M. R. Hasan H. Van Dam N. M. Cortesero A. M. (2013). Belowground induction by *Delia radicum* or phytohormones affect aboveground herbivore communities on field-grown broccoli. Front. Plant Sci. 4:305. 10.3389/fpls.2013.00305 23970888 PMC3748748

[B11] Ryalls J. M. W. Riegler M. Moore B. D. Lopaticki G. Johnson S. N. (2013). Effects of elevated temperature and CO2 on aboveground-belowground systems: a case study with plants, their mutualistic bacteria and root/shoot herbivores. Front. Plant Sci. 4:445. 10.3389/fpls.2013.00445 24273544 PMC3822287

[B12] Shavit R. Ofek-Lalzar M. Burdman S. Morin S. (2013). Inoculation of tomato plants with rhizobacteria enhances the performance of the phloem-feeding insect *Bemisia tabaci*. Front. Plant Sci. 4:306. 10.3389/fpls.2013.00306 23964283 PMC3741575

[B13] Van Geem M. Gols R. Van Dam N. M. Van Der Putten W. H. Fortuna T. Harvey J. A. (2013). The importance of aboveground-belowground interactions on the evolution and maintenance of variation in plant defence traits. Front. Plant Sci. 4:431. 10.3389/fpls.2013.00431 24348484 PMC3842511

[B14] Vannette R. L. Hunter M. D. Rasmann S. (2013). Arbuscular mycorrhizal fungi alter above- and below-ground chemical defense expression differentially among Asclepias species. Front. Plant Sci. 4:361. 10.3389/fpls.2013.00361 24065971 PMC3776932

[B15] Vos I. A. Verhage A. Schuurink R. C. Watt L. G. Pieterse C. M. J. Van Wees S. C. M. (2013). Onset of herbivore-induced resistance in systemic tissue primed for jasmonate-dependent defenses is activated by abscisic acid. Front. Plant Sci. 4:539. 10.3389/fpls.2013.00539 24416038 PMC3874679

[B16] Wurst S. (2013). Plant-mediated links between detritivores and aboveground herbivores. Front. Plant Sci. 4:380. 10.3389/fpls.2013.00380 24069027 PMC3781341

